# Gonadoblastoma in a patient with 45,X/46XY mosaicism

**DOI:** 10.3332/ecancer.2023.1613

**Published:** 2023-09-12

**Authors:** Mercedes Bravo-Taxa, Luis Taxa-Rojas

**Affiliations:** 1 Pathology Department, National Institute of Neoplastic Diseases (INEN), Lima 15038, Peru; 2 Pathology Department, Taxa Oncological Laboratory (LOT), Lima 15038, Peru; 3 University of San Martín de Porres, Lima 15024, Peru; a https://orcid.org/0000-0002-6965-4841

**Keywords:** gonadoblastoma, Turner syndrome, mosaicism, virilisation

## Abstract

45,X/46,XY mosaicism is a sex development disorder with an estimated incidence of less than 1 in 15,000 live births. Various studies have shown there is an increased risk of germ cell tumours forming in Mosaic Turner syndrome. This includes gonadoblastoma, a clinically benign mixed germ-stromal cell tumour. However, this can later develop into one or several malignant germ cell neoplasms, for which early prophylactic gonadectomy is often recommended in patients with 45,X/46,XY mosaicism. The study presents the case of an 11-year-old patient diagnosed with a Mosaic Turner syndrome karyotype, who underwent prophylactic bilateral gonadectomy.

## Introduction

45,X/46,XY mosaicism is a sex development disorder, and those affected by this chromosomal anomaly are at increased risk of developing germ cell tumours. Pre-malignant lesions include intra-tubular germ cell neoplasia and gonadoblastomas (GBs), while the malignant tumours that can form include dysgerminoma, seminoma and non-seminomatous tumours [1–3].

It is estimated that 15% to 20% of patients with 45,X/46,XY mosaicism develop pre-malignant and/or malignant tumours [3]. When the risk of malignancy is compared with the potential gonadal function, in other words, hormone production and future fertility, prophylactic gonadectomy may be indicated due to the increased risk of gonadal tumours [3].

The study presents the case of an 11-year-old patient with 45,X/46,XY mosaicism who underwent prophylactic bilateral gonadectomy due to a high risk of developing a germ cell tumour.

## Clinical case description

An 11-year-old patient diagnosed with a Mosaic Turner syndrome karyotype, who following paediatric, endocrine and gynaecological assessment and monitoring, underwent prophylactic bilateral gonadectomy.

**Figure 1.**
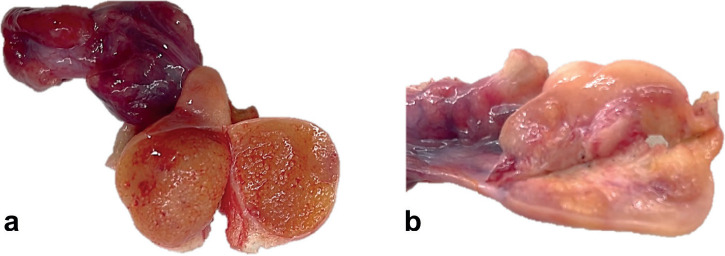
(a): Right abdomen: ovary replaced by yellow-brown granular parenchyma, resembling or simulating testicular parenchyma. (b): Left abdomen: ovary with yellow-brown granular nodular areas.

Macroscopically, the right abdomen comprised a congestive fallopian tube and ovary (1.5 × 1.0 × 1.0 cm) replaced by a yellow-brown granular surface that resembled or had macroscopic characteristics similar to a testicle. Likewise, the left abdomen comprised a congestive fallopian tube and ovary with yellow-brown granular nodules similar to the tumour on the right side (Figure 1).

Microscopically, both ovaries showed diffuse proliferation of nests of variable size containing two cell populations: germ cells and sex cord cells, the latter located on the periphery of the nests, which were also embedded in variable amounts of fibromatous stroma with Leydig/luteinised cells. Further, a variable amount of basal membrane-like material was observed inside some nests.

**Figure 2.**
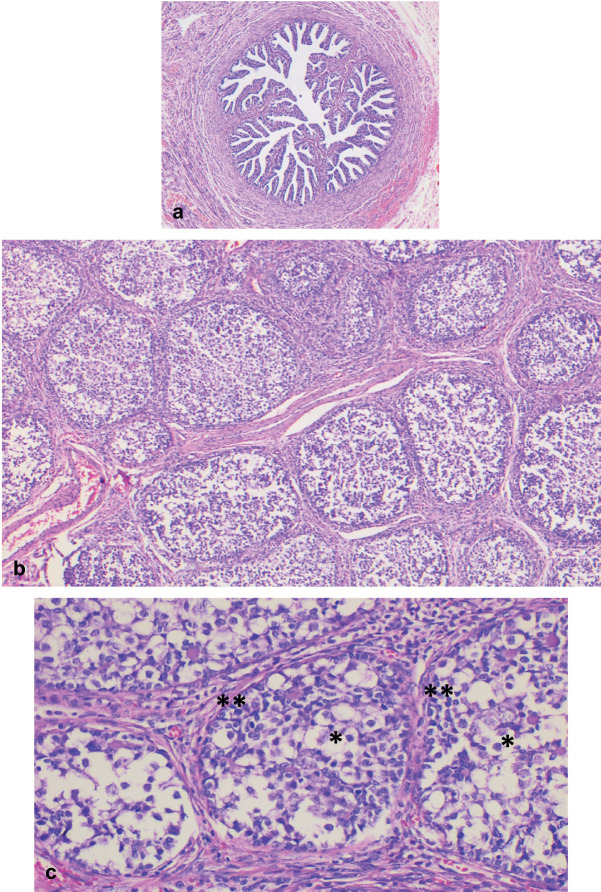
Right abdomen. (a): Congestive fallopian tube. (b): Ovary with proliferation of nests of different sizes, separated by a variable amount of fibromatous stroma. (c): Nests comprising two cell populations: germ cells in the central portion (*), and sex cord cells along the periphery (**).

**Figure 3.**
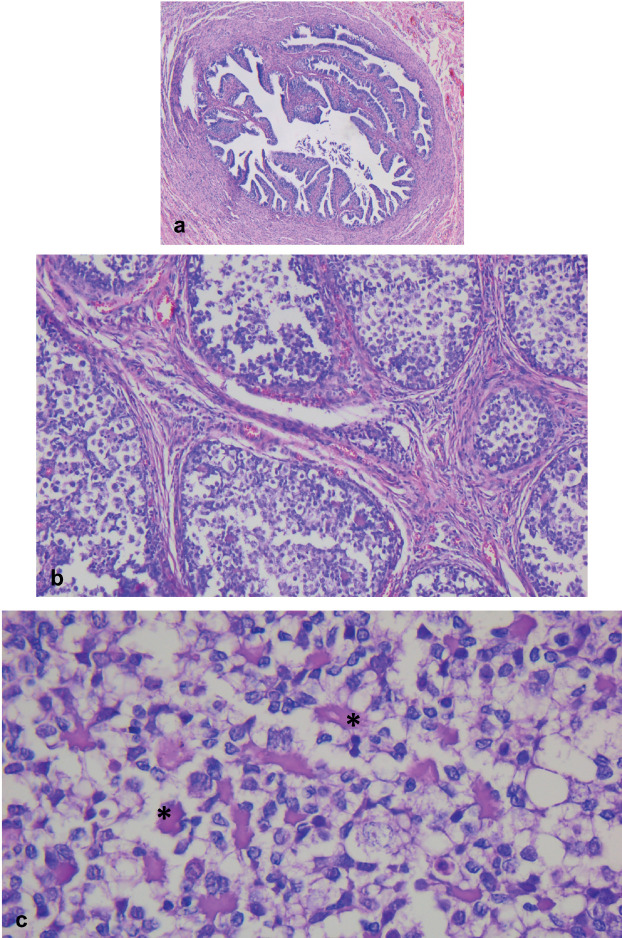
Left abdomen. (a): Congestive fallopian tube. (b): Ovary with a proliferation of nests of different sizes comprising two populations of tumour cells, separated by a variable amount of fibromatous stroma. (b): Basal membrane material can be identified within the nests (*).

The cytological characteristics of the two tumour cell populations were: germ cells with abundant, pale cytoplasm; round, large nucleus; and often, prominent nucleolus. Meanwhile, the sex cord cells presented with scant cytoplasm; small nucleus; and inconspicuous nucleolus (Figures 2 and 3).

**Figure 4.**
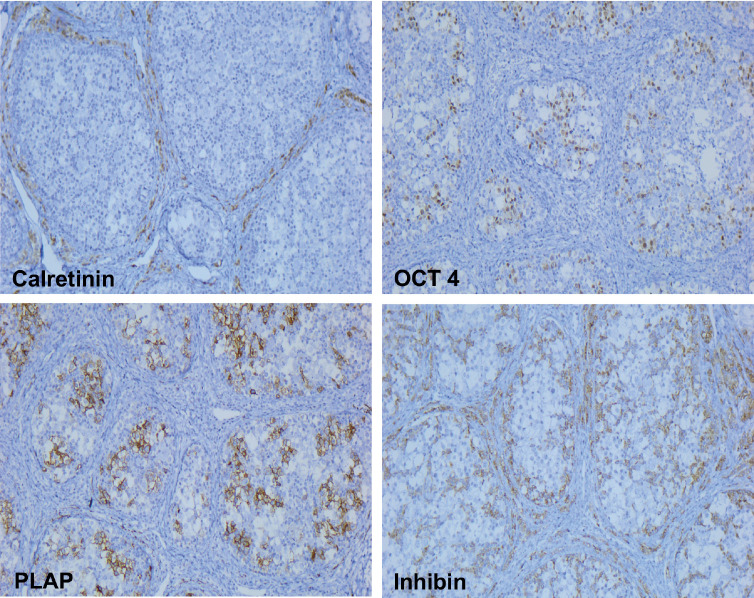
Immunohistochemistry: the germ cells were positive for PLAP and OCT4 while the sex cord and Leydig/luteinised cells were positive for inhibin. In contrast, the germ cells as well as the sex cord cells were negative for calretinin.

The immunohistochemical profile was positive for placental-like alkaline phosphatase (PLAP) and Octamer biding transcription factor 4 (OCT4) in the germ cells, and positive for inhibin in the sex cord and Leydig/luteinised cells. While both tumour cell populations were negative for calretinin, the Leydig/luteinised cells did immunoexpress it (Figure 4).

With all of these morphological and immunohistochemical characteristics in conjunction with the history of Turner Mosaic syndrome, it was decided that the patient also suffered from bilateral GB, affecting both ovaries.

## Discussion

The most common sex chromosome anomaly in women is Turner syndrome, with an incidence of one in 2,000–2,500 live female births. Six to nine per cent of girls with Turner syndrome have a mosaic karyotype; in other words, a structurally anomalous Y chromosome or fragments of Y chromosome material [2]. 

Mosaic Turner syndrome with Y chromosome-derived material (MTSY) has an estimated incidence of less than 1 in 15,000 live births [3]. 

The resulting phenotype varies significantly, and can range from external female genitalia with striated gonads, to external male genitalia with testicles. Affected individuals may also have ambiguous external genitalia with dysgenetic testicles, or one dysgenetic testicle and one contralateral streaked gonad [4]. No correlation has been found between the phenotype and the level of karyotypic mosaicism [5].

MTSY also increases the risk of developing GB which, may lead to the development of one or more malignant germ cell neoplasias [6, 7].

GB is a clinically benign mixed germ-stromal cell tumour comprising closely intermingled sex cord cells and germ cells, adopting a distinctive nest-like pattern. However, owing to its predisposition to develop into a malignant germ cell neoplasia, early prophylactic gonadectomy is often recommended. However, the optimal timing for surgery is unclear [2, 8]. The major limit and delay in performing surgery are the family’s concerns, and the lack of information about this pathology for the family and patient. Therefore, making the decision requires multidisciplinary medical advice and support; particularly psychological support for the patient and genetic counselling for the family.

Furthermore, knowing the increased risk that GB presents in developing a malignant germ cell neoplasia, prophylactic gonadectomy may be indicated. For the family and the patient, in conjunction with the medical team, the risk of malignancy must be taken into account when making the decision, and weighed against the preservation of gonadal function, hormone production and future fertility.

GB develops almost exclusively in dysgenetic gonads in those with mosaicism with Y chromosome material, and in those with pure gonadal dysgenia [8]; furthermore, these constitute two-thirds of all gonadal tumours [9].

The general risk of developing GB in MTSY is approximately 15%–30%; however, it depends on age, ranging from 3% to 4% at 10 years to 46% at 40 years [3, 7]. However, the fact that GB occurs more frequently in the second decade of life may be due to a late diagnosis of MTSY, which in turn leads to a later prophylactic gonadectomy [2]. 

It typically affects children and young adults, with the majority of these detected prior to 15 years of age. Matsumoto *et al* [9] reported on a group of seven women with MTSY, aged 2–11 years, four of whom developed GB.

On macroscopic exam, GB often presents with a solid appearance, yellow to greyish in colour. In other instances, the cut surface may be sandy, or soft and fleshy, or firm and cartilaginous, with calcified areas or completely calcified. Tumours are typically small (<3 cm), bilateral, and the underlying gonad is nearly always dysgenetic [10]. This case report presents tumours in both ovaries, the largest diameter measuring <2.0 cm.

GB is characterised by its composition of nests of various sizes comprising germ cells and primitive sex cord cells embedded in fibromatous stroma with Leydig/luteinised cells. Infiltrative or diffuse growth patterns are seen less commonly. In some cases, they may mimic a germinoma when there are low numbers of sex cord cells. On the other hand, calcifications are common, ranging from small dots to complete replacement by irregular, calcified masses. GB nests typically display germ cells with abundant, pale cytoplasm; large, round nuclei; and often, prominent nucleolus, with different degrees of maturation and presence of mitosis. In contrast, sex cord cells have scant cytoplasm and a small nucleus with an inconspicuous nucleolus; these cells are distributed at the periphery of the nests and surrounding basal membrane-type material which may be variably prominent within the nests [10]. All of these characteristics were found in both ovaries in the present case report, with the exception of the presence of calcifications.

Immunophenotypic expression typically presents in germ cells positive for POU5F1, SALL4, D2-40, OCT3/4, C-kit and podoplanin, while the sex cord cells and Leydig/luteinised cells are positive for inhibin, FOXL2, calretinin, SF1, SOX9, CD56 and may be positive for WT1 and AE1/3 [11]. 

The prognosis is excellent, if pure. However, it may be differentiated into germ cell tumours, such as dysgerminomas, and, less frequently, teratomas, embryonal carcinoma, vitelline sac tumours and choriocarcinoma. The presence of malignant germ cell tumours is that which changes the prognosis, depending upon the type and extent [11]. It has been noted that unregulated growth in the germ cell component of GB leads to dysgerminoma or seminoma in approximately 35% of cases [12].

## Conclusion

- 45,X/46,XY mosaicism is a sex development disorder.
- Patients with 45,X/46,XY mosaicism have a higher risk of developing germ cell tumours.
- GB is a clinically benign, mixed germ cell-stromal tumour.
- GB has a predisposition to develop into a malignant germ cell neoplasia.
- The benefit of prophylactic gonadectomy must be weighed against the preservation of gonadal function and fertility.
- To alleviate casuistry and improve knowledge of this disease, more similar cases will be compiled with a goal of determining the incidence germ cell neoplasia developing from GB, the most frequently found histological type of germ cell neoplasia, and having improved evaluation and decision making regarding the opportune moment to perform a prophylactic gonadectomy.

## List of abbreviations

GB, Gonadoblastoma; MTSY, Mosaic Turner syndrome with Y-chromosome-derived material.

## Conflicts of interest

The authors declare no conflict of interest regarding the publication of this case report.

## Funding

This publication has not received any specific subsidies or funding from public, commercial or non-profit agencies.

## Author contributions

Dr Bravo analysed the clinical data, wrote the paper and did the final editing. Dr Taxa provided the pathological findings, provided professional guidance, and was instrumental in the critical review of this study. All authors have read and approved the final edition of this paper.
